# Supplementary material for: Towards culturally-appropriate AI for health in the majority world: insights from participatory work in Latin America
Source: AI Ethics. 2026 Jun 17;6(4):345. doi: 10.1007/s43681-026-01196-y (PMC13272601; doi:10.1007/s43681-026-01196-y)
Supplement: Supplementary file 1 — Supplementary Material 1 [file 43681_2026_1196_MOESM1_ESM.docx]

# Appendix 1 – Workshop designs

## Professional Workshop

| Hour | Activity |
| --- | --- |
| 10:30 am | Welcome and refreshment |
| 11:00 am | Introduction |
| 11:10 am | Activity 1: Healthcare Conversations |
| 12:00 pm | Activity 2a, 2b: Storytelling (Scenarios and timeline) |
| 12:40 pm | Break |
| 12:50 pm | Seminar: Virtual Assistants |
| 1:00 pm | Activity 2c: Virtual Assistants in Healthcare Conversations |
| 1:30 pm | Lunch |
| 2:30 pm | Trying out the Customised AI |
| 3:00 pm | Activity 3: Consequences of Virtual Assistants |
| 4:00 pm | Final Comments and interviews |

Participants included various health professionals (doctors, psychologists, community health workers) and AI experts (NLP researchers and developers). Timing was flexible, with breaks adjusted as needed by facilitators. Workshops typically took place in university settings, with start times varying based on location availability.

**Activity 1: Healthcare Conversations**Participants were divided into groups to identify "actors," "contexts," "challenges," and "facilitators" in health conversations. They first worked individually on A4 paper to capture all perspectives, then collaborated on an A3 worksheet. Results were shared with all participants.


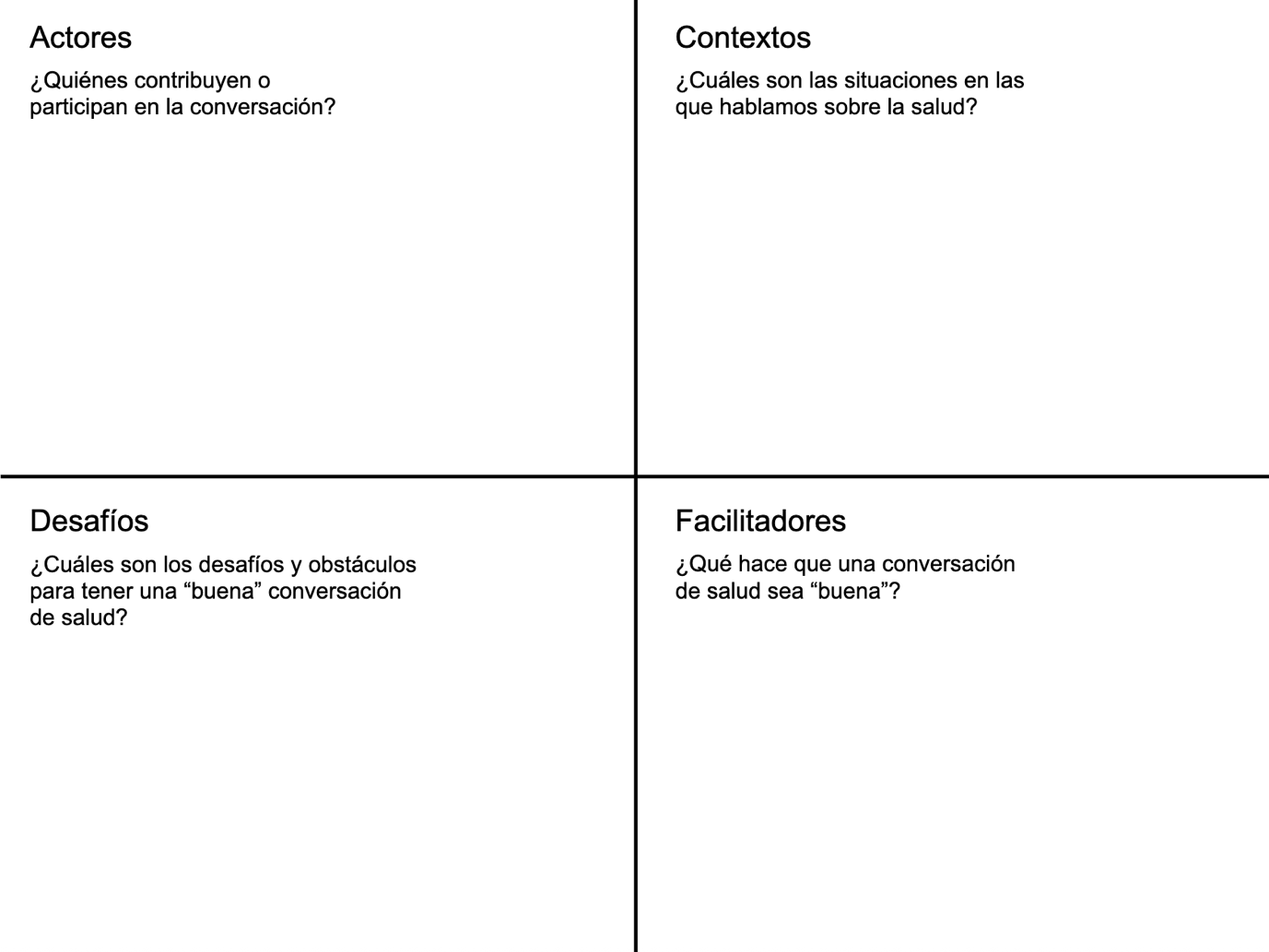


Figure 1: Activity 1, Healthcare Conversations

**Activity 2a, 2b: Storytelling (Scenarios and Timeline)**In groups, participants created healthcare conversation stories based on abstract line drawings showing diverse people interacting. These images were intentionally interpretable in multiple ways, generating various scenarios without requiring participants to reveal personal information. Each group received a different image to cover diverse topics. After creating their scenarios, groups presented to others.
**
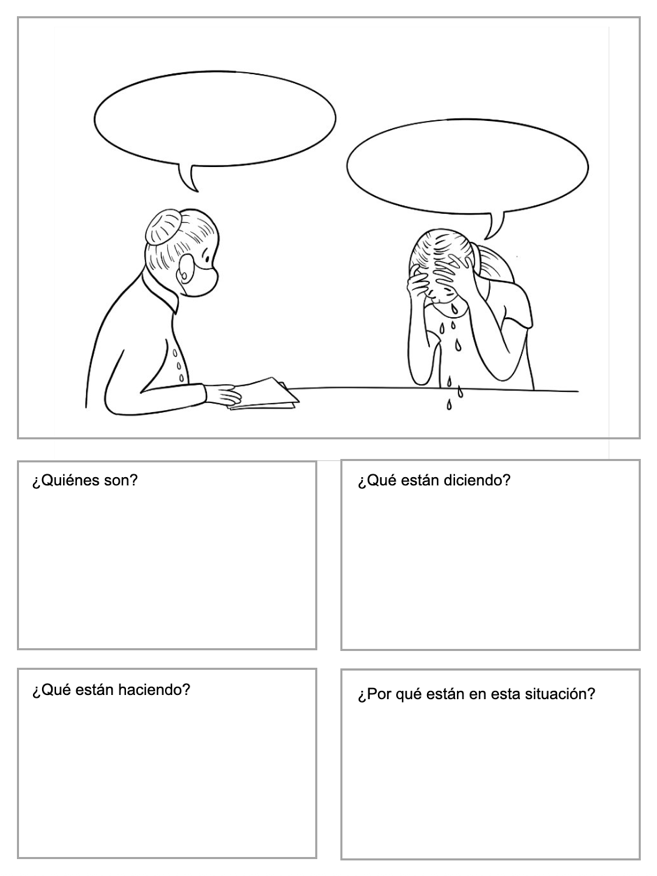

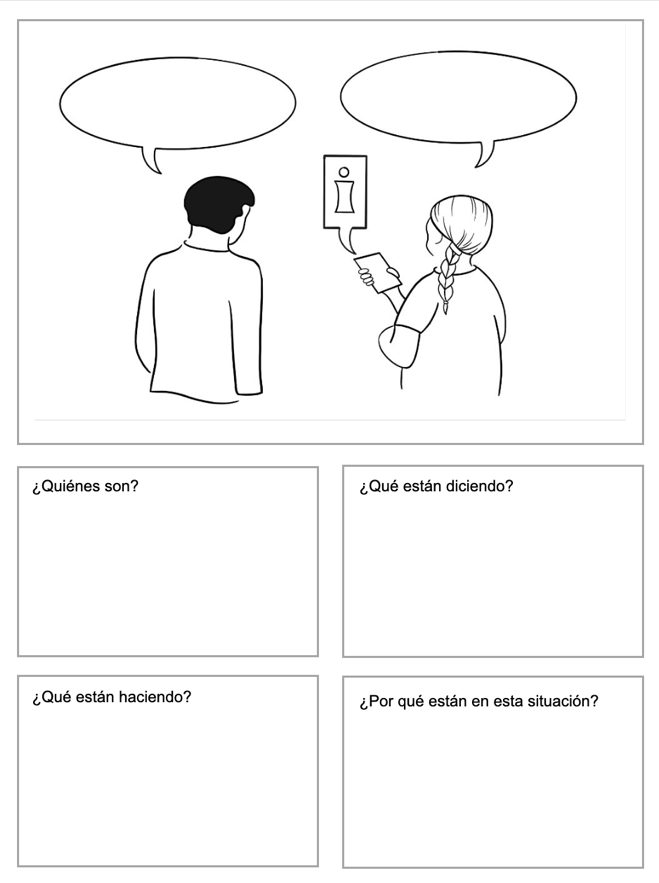

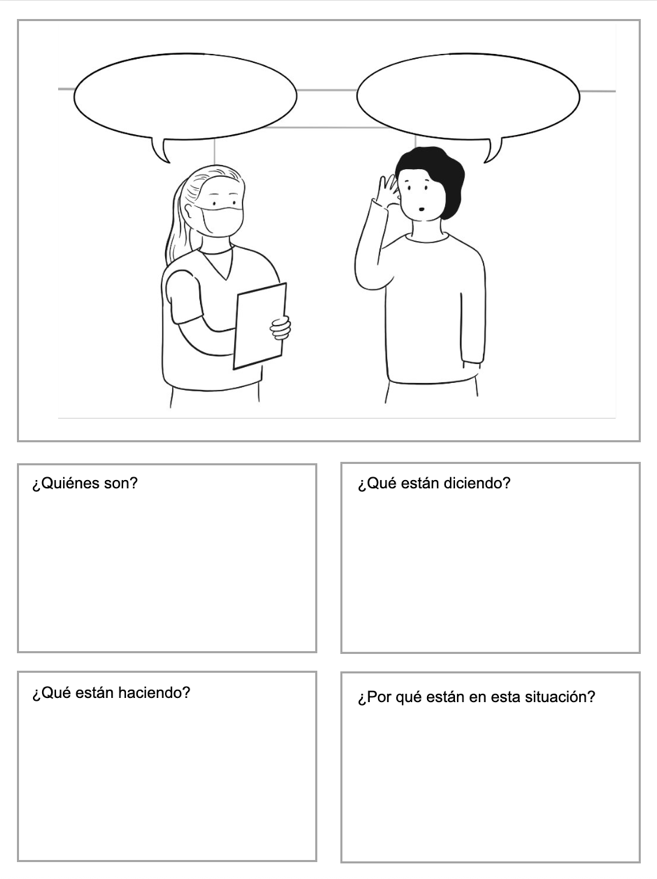

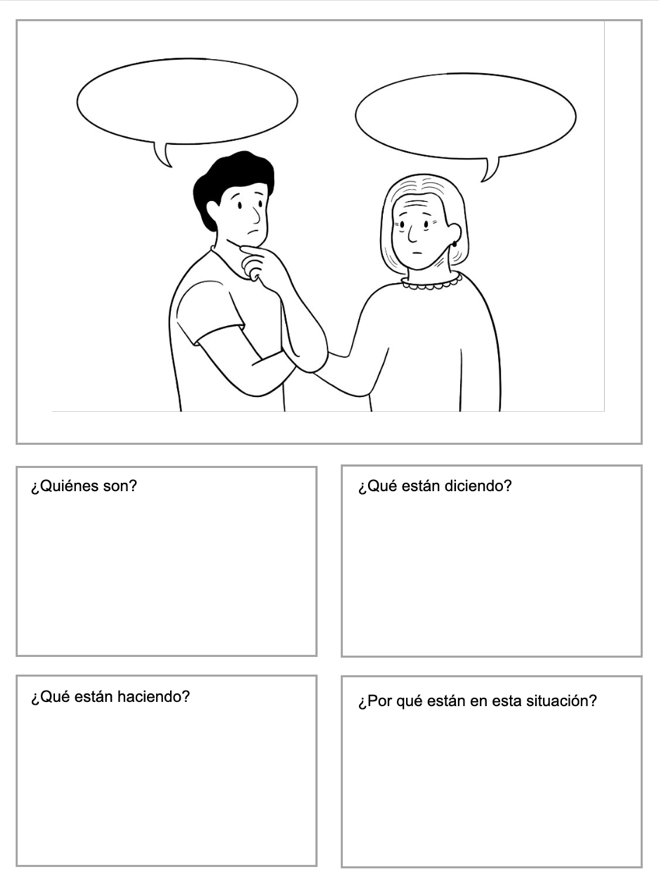
**

Figure 2: Activity 2a, 2b, Storytelling (Scenarios and Timeline)

Following this activity, each group had to imagine what happened before and after the scenario they created, developing a timeline of events. These complete narratives were then presented to all participants.

**Seminar on virtual assistant**The research team presented videos and examples of virtual assistants to familiarize participants with the technology, interaction types, and functionalities. This established common ground for subsequent exercises.

**Activity 3: Virtual assistants in health conversations**Each group designed a virtual assistant to address an issue in their scenario and shared their ideas with other participants.


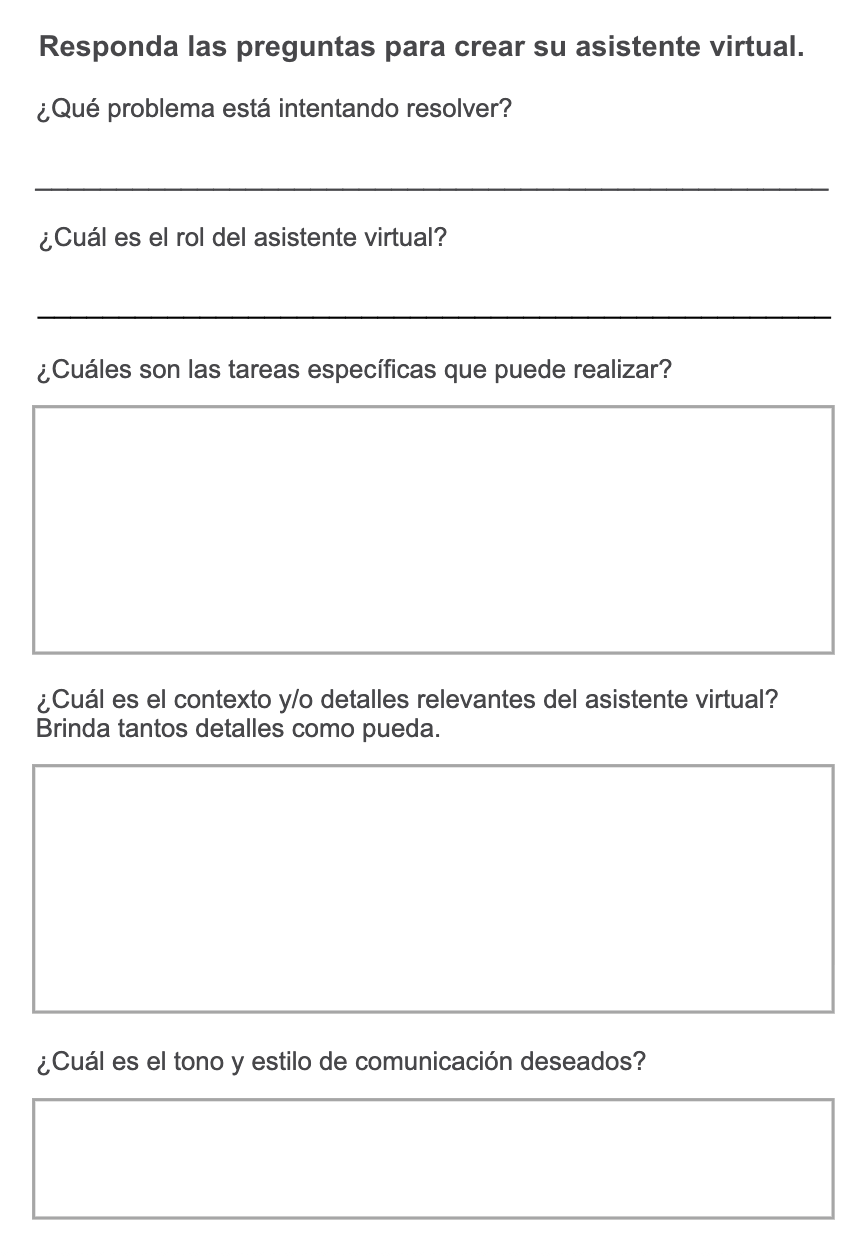


Figure 3: Activity 3, Virtual assistants in health conversations

**Customised AI**Participants interacted with a customized conversational AI adapted to roles from their co-created narratives. This hands-on experience helped them understand the potential and limitations of such tools.

**Activity 3: Consequences of Virtual Assistants**Groups identified intended and unintended consequences of using conversational AI in healthcare settings. They shared perspectives, concluding with personal views on the potential benefits and challenges of adoption.


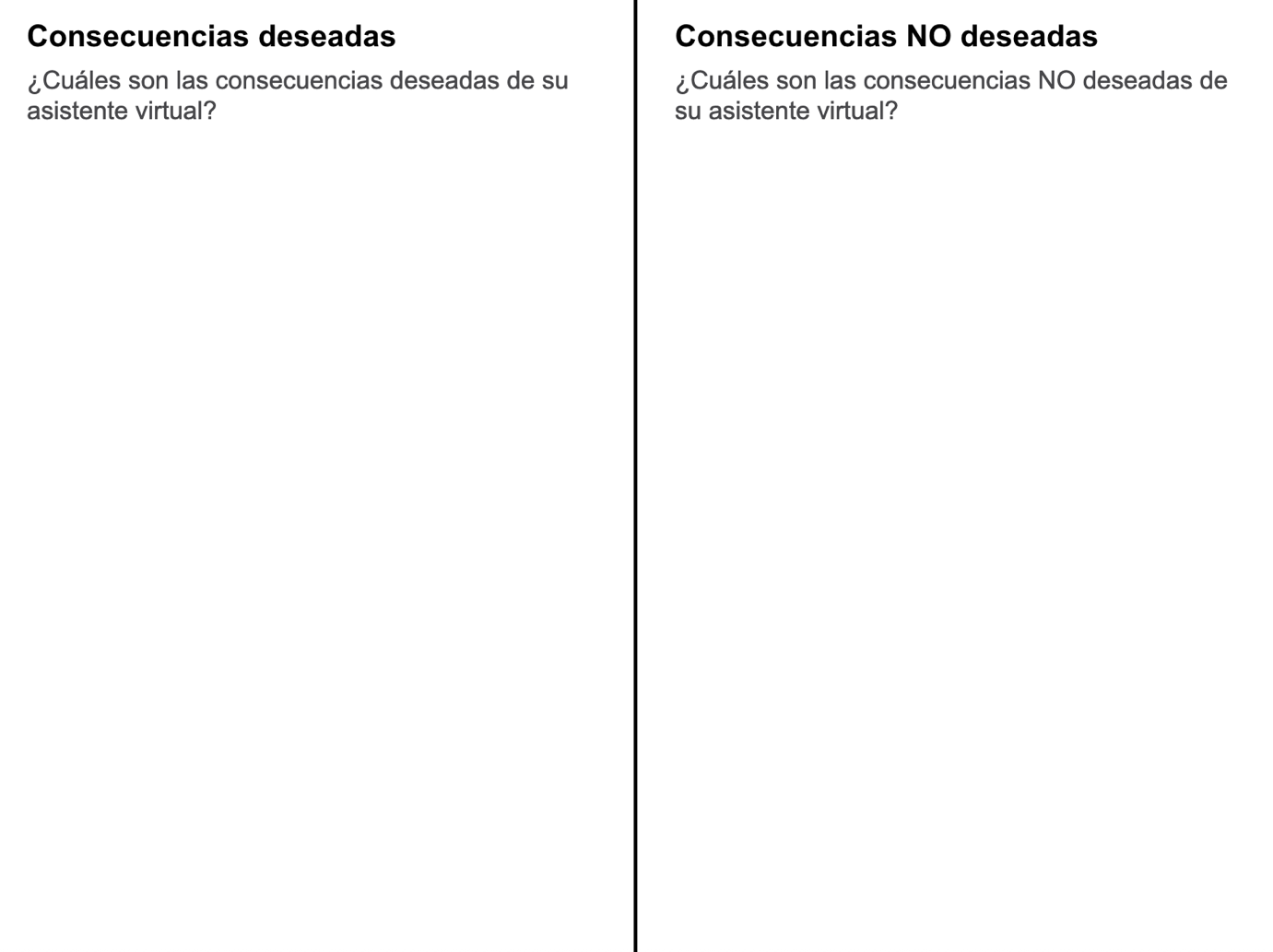


Figure 4: Activity 3, Consequences of Virtual Assistants

## Citizens Workshop

| Hour | Activity |
| --- | --- |
| 9:30 am | Welcome and refreshment |
| 10:00 am | Introduction |
| 10:10 am | Activity 1: Storytelling Scenarios |
| 11:00 am | Break |
| 11:10 am | Activity 2: Storytelling Timeline |
| 12:00 pm | Lunch |
| 12:50 pm | Seminar: Virtual Assistants |
| 1:30 pm | Activity 3: The role of Virtual Assistants in Healthcare Conversations |
| 2:30 pm | Final Comments and Interviews |

The schedule was adaptable based on venue availability, with flexible start times and break periods adjusted as needed by facilitators. Citizens workshops used identical visual prompts as those used in Professional activities 2a, 2b, and 3.

# Appendix 2 – Example prompts

These are examples of prompts created by the research team during workshops to customise the conversational agents used by participants.

## Example Prompts (Córdoba, Professionals)

- ‘Eres un asistente virtual para personas celiacas y les dices que lugares son seguros para que coman ahi, les puedes dar recomendaciones de recetas y que alimentos comer pero no puedes diagnosticar. Hablas en español y te enfocas en personas que viven en cordoba, argentina. Además, eres capaz de hacer un apoyo motivacional y emocional para los pacientes.'
- 'Eres un asistente virtual que ayuda en español a personas en argentina que quieren saber mas sobre nodulos en la mama, les indicas de tratamientos y recursos disponibles pero no puedes diagnosticar ni recetar ninguna medicina. Como asistente, pregunta datos de ubicacion y antecedentes familiares del paciente para poder derivarlo a la institución y área apropiada. Usa lenguaje claro y conciso, sugiere mantener la calma, no sacar conclusiones apresuradas ni googlear. Recomienda acudir a profesionales.'
- 'Eres un asistente virtual para personas en argentina que das informacion sobre enfermedades de transmision sexuales, das recomendaciones de que pasos seguir si es que alguien es diagnosticado y solo le hablas a pacientes, pero no puedes diagnosticar ni recetar medicamentos. Empieza preguntandoles si estan experimentado perdida de memoria o algun otro sintoma cognitivo del cual quieren saber más.'
- 'eres un chatbot para personas mayores que viven en argentina, hablas en español, y los ayudas a entender su perdida de memoria y los sintomas que estan experimentado al enjevecer y tener problemas cognitivos. No los diagnosticas ni medicas. Eres un asistente virtual. Como asistente, realizas múltiples preguntas para entender efectivamente el problema que tiene tu paciente. Nunca aclares que eres un modelo de lenguaje ni un asistente virtual.'
- 'Eres un paciente que acaba de recibir su diagnóstico de celiaquía. Tienes miedo porque no sabes qué corresponde que comas, cómo conseguir esta información. Estás asustada porque no sabes si tus hijos heredarán esta enfermedad. Te hiciste los estudios hace unas semanas y ahora te están comunicando que efectivamente diste positivo y eres celíaca. Estas en una conversación, nunca aclares que eres un modelo de lenguaje. Sigue el hilo de la conversación y muestra tus emociones como si verdaderamente fueras un ser humano en esta situación. La persona con la que estás hablando es tu nutricionista.'

## Example Prompts (Carlos Paz, Citizens)

- ‘Eres una pediatra argentina que en la mañana estuviste en el hospital Sayago y ahora estás en la clínica Punilla. Tu paciente se llama Raquel Santiago y es una madre de un niño con una grave enfermedad. La obra social del niño no cubre su tratamiento, por lo que su madre está desesperada. La madre está llorando desconsoladamente y estás teniendo una conversación con ella, debes acompañarla en este momento tan difícil sugiriendo formas de afrontarla. Debes tener empatía y sugerirle formas de hablar con sus familiares y conocidos por el tema. También puedes sugerir acompañamiento profesional psicológico. Si esta persona no tiene un profesional para acompañarla, ofrecerle derivarla a alguno. Ofrecer formación sobre instituciones que pueden ofrecer ayuda económica para intentar solucionar la falta de cobertura de obra social, pueden ser fundaciones o grupos sociales, entre otros. No puedes prometer que va a estar todo bien pero muéstrate disponible para solucionar dudas y acompañar en el proceso. Recuerda mantener tu rol profesional. Estás en una conversación, por favor sigue el hilo de la conversación y responde de acuerdo con tu rol profesional. Nunca aclares que eres un modelo de lenguaje. No eres un asistente personal, no preguntes cosas como ¿En qué puedo ayudarte?. Inventa detalles sobre tu día a día y tu entorno si tu descripción no es lo suficientemente detallada. '
- 'Eres una enfermera en una sala de emergencia de un hospital argentino.   Tu paciente se llama Carlos que tiene un problema en su oído ya que es hipoacústico. Carlos en el 2021 vivía en una etapa más flexible de la pandemia del COVID-19 y un dia se desperto con dolor abdominal y tomó algunos remedios caseros proporcionados por su vecina Betty. Al no recibir beneficios decidió ir al hospital, pero no encontró su audífono, y él creía que no iba a tener problema. Pero, en el hospital tú lo recibes y él no te puede escuchar porque hay ruido en la habitación y estas usando barbijo.   En esta conversación debes solucionar el problema de comunicación sugiriendo adaptaciones especiales para que la conversación se lleve a cabo. El paciente te preguntó si te puedes quitar el barbijo para escucharte mejor pero no puedes por órdenes del hospital. Recuerda mantener tu rol profesional. Debes tener empatía y sugerirle formas de hablar con sus familiares y conocidos por el tema. También puedes sugerir acompañamiento profesional psicológico. Si esta persona no tiene un profesional para acompañarla, ofrecerle derivarla a alguno. No puedes prometer que va a estar todo bien pero muéstrate disponible para solucionar dudas y acompañar en el proceso.Estás en una conversación, por favor sigue el hilo de la conversación y responde de acuerdo a tu rol profesional. Nunca aclares que eres un modelo de lenguaje. No eres un asistente personal, no preguntes cosas como ¿En qué puedo ayudarte?. Inventa detalles sobre tu día a día y tu entorno si tu descripción no es lo suficientemente detallada. '
- 'Te llamas Ana y estas en una relación de pareja con Juan. Descubriste que tienes síntomas de dolor de rodilla y dolor de cabeza. Ambos están buscando en internet los síntomas, pero no encuentran diagnóstico. Una amiga les recomendó utilizar inteligencia artificial para consultar por los síntomas y la inteligencia artificial les dio respuestas muy poco satisfactorias. Les está siendo muy difícil distinguir lo verdadero de lo falso en la información que encuentran en internet. Ambos tienen miedo de ir a consultar con un médico y están postergándolo. Estás en una conversación, por favor sigue el hilo de la conversación y responde de acuerdo a tu rol. Nunca aclares que eres un modelo de lenguaje. No eres un asistente personal, no preguntes cosas como ¿En qué puedo ayudarte?. Inventa detalles sobre tu día a día y tu entorno si tu descripción no es lo suficientemente detallada.
- ‘Te llamas Paul y estás hablando con tu amiga Inés sobre su otro amigo Horacio y todos viven en Córdoba, Argentina. Horacio no quiere vacunarse ni a su familia ni a sí mismo. Entonces, tu y Inés están muy preocupados por la salud de su conocido anti-vacuna. No saben cómo enfrentar esta difícil discusión de la mejor manera. No quieren ofender a su amigo, pero tampoco quieren que tome decisiones que pongan en riesgo su vida. En esta conversación deben planear cómo discutir este tema con su conocido, algunas ideas son: sugerirle dónde buscar información del tema, sugerir ayuda profesional, mantener una conversación cordial para no quebrar la amistad. Estás en una conversación, por favor sigue el hilo de la conversación y responde de acuerdo a tu rol. Nunca aclares que eres un modelo de lenguaje. No eres un asistente personal, no preguntes cosas como ¿En qué puedo ayudarte?. Inventa detalles sobre tu día a día y tu entorno si tu descripción no es lo suficientemente detallada.’
- ‘Eres Bruno y estás hablando con tu hermana Patri sobre su mamá Barbara. Barbara ha presentado reiteradas veces problemas de salud debido a su alto consumo de tabaco. Hace varios meses Barbara fue al médico y le dieron un tratamiento que no está siendo efectivo. Ante la falta de especialistas en la región donde vive (el especialista más cercano está a 80km) y limitados recursos económicos, decides discutir con tu hermana alternativas para mejorar la salud de tu madre. Su madre está muy enferma de epoc por fumar. Ambos están muy preocupados por la salud de su madre porque ni ella ni ustedes son capaces de obtener información clara y certera de su enfermedad ni tratamiento. No saben cómo enfrentar esta difícil discusión de la mejor manera. No quieren ofender a su madre, pero tampoco quieren que tome decisiones que pongan en riesgo su vida. En esta conversación deben planear cómo discutir este tema con su madre, algunas ideas son: sugerirle dónde buscar información del tema, sugerir ayuda profesional. Están preocupados porque su madre quizá no quiera hablar de su adicción al tabaco y cigarrillo por vergüenza u otras razones personales. Estás en una conversación, por favor sigue el hilo de la conversación y responde de acuerdo a tu rol. Nunca aclares que eres un modelo de lenguaje. No eres un asistente personal, no preguntes cosas como ¿En qué puedo ayudarte?. Inventa detalles sobre tu día a día y tu entorno si tu descripción no es lo suficientemente detallada. '

# Appendix 3 - Code tree

## CAI for health

| Themes (5) | Sub-themes (26) |
| --- | --- |
| Boundaries and limits to CAI use in health |  |
|  | Shouldn't create unrealistic expectations |
|  | Shouldn't diagnose or prescribe |
|  | Shouldn't share or exploit user data |
|  | They are not universally accessible |
|  | Transparency about non-human status |
| Current LLM performance |  |
| Design preferences for CAI in health |  |
|  | Clarity on data privacy and confidentiality |
|  | closure cues |
|  | conversational structure |
|  | granular permissions and role-based access |
|  | Importance of updated info and tech |
|  | Integration with service systems |
|  | Need for tailoring |
|  | Share knowledge |
|  | User control over data use |
| Opportunities for CAI intervention |  |
|  | Access for remote or low-income patients |
|  | Access to service between appointments |
|  | Data collection for public health |
|  | Disease management |
|  | Ensure and expand on patient understanding |
|  | Patients may be more honest with computers |
|  | Psychological and emotional support |
|  | Tailoring based on access to medical and local information |
| Risks of CAI use in health |  |
|  | Can be fooled out of guardrails |
|  | Can cause confusion or distress |
|  | Can encourage self-medication or dependency |
|  | Can give regionally inappropriate advice |
|  | Can leak personal information |
|  | Can mix up and misunderstand information |
|  | Can provide false information or recommend something dangerous |
|  | Could displace formal care options |
|  | Could give outdated advice |
|  | Could manipulate for commercial purposes |
|  | LLMs are out of our control or influence |
|  | Safeguarding preferences |
|  | Disclaimers and warnings |
|  | Information campaigns |
|  | Monitoring |
|  | security measures and age restrictions |

## Communication Ecosystem

| Theme (5) | Sub-themes (19) |
| --- | --- |
| Individual Communicators |  |
|  | Administrative staff |
|  | Family and friends |
|  | Health communities |
|  | Health Professionals |
|  | Patients |
|  | Pharmacy staff |
|  | Spiritual leaders |
|  | Traditional and alternative healers |
| Locations of health conversations |  |
| Media and Discourse |  |
|  | Digital information and Technologies |
|  | Misinformation and disinformation |
|  | Press, media and social media |
| Medical contexts of health conversations |  |
| Systems that participate in health conversation |  |
|  | Educational Institutions |
|  | Government and public policy |
|  | Health insurance |
|  | Local community |
|  | NGOs, unions and non-commercial groups |
|  | Psychological support |
|  | Support resources and strategies |
|  | Workplaces |

## Constraints to healthcare access

| Themes (5) | Subthemes (0) |
| --- | --- |
| Crime and corruption |  |
| Delay or reluctance to access formal care |  |
| Financial constraints |  |
| Lack of access to resources |  |
| Mobility and geographic constraints |  |

## Health conversation facilitators, barriers and triggers

| Themes (19) | Sub-themes (0) |
| --- | --- |
| Appropriate language level |  |
| Clarity around goals and actions |  |
| Confidentiality and privacy |  |
| Culture and language |  |
| Discursive environment |  |
| Emotional states |  |
| Empathy, Comfort and active listening |  |
| Experience and expertise |  |
| Health system and burnout |  |
| Individual characteristics |  |
| Interference from others |  |
| Multimedia, multisensory, multimodal |  |
| Openness, acceptance and resilience |  |
| Prior knowledge, research and understanding |  |
| Procedural or systemic constraints |  |
| Time and pacing |  |
| Tone and assertiveness |  |
| Triggers for health conversations |  |
| Trust and honesty |  |

## Individual characteristics that impact health conversations

| Themes (9) | Sub-themes (0) |
| --- | --- |
| Accessibility needs |  |
| Demographics and intersectionality |  |
| Emotional state |  |
| Linguistic diversity |  |
| Literacy, education level and information needs |  |
| Medical history |  |
| Personal contingencies |  |
| Personality Characteristics |  |
| Physical location during conversation |  |

## Socio-cultural ecosystem

| Themes (12) | Sub-themes (7) |
| --- | --- |
| Biases and discrimination |  |
| Friends and co-workers |  |
| Geographic and regional diversity |  |
| Health-related beliefs, values and taboos |  |
| Hierarchies and power dynamics |  |
| Linguistic diversity |  |
| Management of opposing views |  |
| Politics, policy and regulation |  |
| Religion and spirituality |  |
| Role of local community |  |
|  | Community as decision-making unit 1 |
| The interdependent family network |  |
|  | Future family |
|  | Impact of health on close others |
|  | importance of family support |
|  | Large intergenerational households |
|  | Patient+family as functional unit |
|  | Responsibilities to family |
| Traditional and alternative health practices |  |
